# Effect of a Novel Patient Garment on Perceived Privacy during Colonoscopy: A Simple Approach to Minimize Embarrassment

**DOI:** 10.1155/2019/2467101

**Published:** 2019-01-27

**Authors:** Ali Aamar, Zeeshan Butt, Kamraan Madhani, Iqra Hussain, Joel Garsten, Harry Aslanian

**Affiliations:** ^1^Gastroenterology & Hepatology Fellow, The Brooklyn Hospital Center, 121 DeKalb Avenue, Brooklyn, NY 11201, USA; ^2^PG-3 Prince George's Hospital, Cheverly MD, USA; ^3^Yale-Waterbury Internal Medicine Residency Program, Yale New Haven Hospital Waterbury, USA; ^4^Department of Public Health, Waterbury, CT, USA; ^5^Yale School of Medicine, Waterbury Hospital, USA; ^6^Division of Gastroenterology, Yale New Haven Hospital, 40 Temple Street, New Haven, CT, USA

## Abstract

**Background:**

In the United States, patients wear a one-piece, reusable cloth gown during colonoscopy procedures. Many patients report embarrassment related to bodily exposure during colonoscopy. This may limit participation in colorectal cancer screening programs.

**Aims:**

To assess whether the use of a novel, disposable patient garment (Privacy Pants, Jackson, MS), which increases patient coverage, can reduce embarrassment related to bodily exposure and increase colonoscopy acceptance rates.

**Methods:**

Patients were offered a novel gown, and they completed questionnaires before and after colonoscopy.

**Results:**

A total of 120 patients participated. 54% were female and 82% were Caucasian. The novel gown had high overall satisfaction (8.3) and was associated with a sense of respect during the procedure (9.4). 67% (80) of the patients had a prior colonoscopy, and of these, 76% would request a novel gown over a traditional gown for future procedures. Among all study participants, a high rate of acceptability for repeat colonoscopy if recommended by their doctors was reported (mean of 9.4). Nonwhites were more likely to have a concern for embarrassment addressed by using novel gowns as compared to whites (*P* value 0.02).

**Conclusion:**

All participants, particularly women and nonwhite participants, reported high rates of respect and satisfaction and decrease in embarrassment utilizing the novel gown during colonoscopy. Patients who had prior colonoscopy with a traditional gown preferred the novel garment. A novel procedure gown may enhance colonoscopy acceptance by minimizing embarrassment.

## 1. Introduction

**Figure 1:**
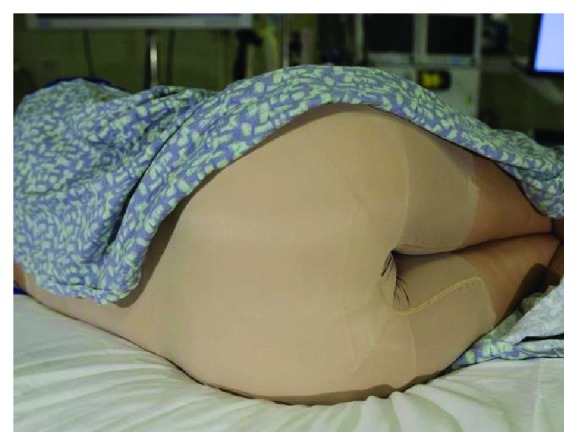
A model patient wearing a traditional back-opening hospital gown before and during colonoscopy (reprinted with permission from Dignity Garment LLC).

Approximately 15 million colonoscopy procedures are performed annually in the United States, most commonly as an outpatient procedure. The typical current practice in the United States (US) is for patients to wear a one-piece, reusable cloth gown (Figure 1). The gown is typically knee high with a tie in the back that may provide limited coverage of patients' back. Patients may feel vulnerable and anxious about bodily exposure during colonoscopy [1–5]. Patients typically are placed in the left lateral position for colonoscopy, and their buttocks are exposed. Occasionally, patients need to be turned supine, prone, or right lateral to facilitate scope passage with limited coverage of pelvic anatomy with position changes. Bodily exposure may also be uncomfortable for endoscopy staff.

Currently, colon cancer screening rates in the United States are approximately 65% [6]. Embarrassment is a significant barrier to colonoscopy screening [7]. This is highlighted by the frequent preference of female patients for a female colonoscopist [8]. A large majority of GI endoscopists in the US, however, are male [9, 10]. Members of lower socioeconomic groups are less likely to receive cancer-preventive services [11]. Traditional patient gowns may be associated with a loss of dignity, the reinforcement of the “patient role,” and the assumption of a low-status position in the hospital [12]. In addition, lack of protection for patient privacy could create potential medicolegal issues [13, 14]. A prospective study was performed to evaluate the impact of a novel privacy pant garment on patient's perception of privacy.

## 2. Methods

This is a cross-sectional clinical study conducted at an outpatient, hospital-affiliated surgical center (Naugatuck Valley Surgical Center in Waterbury, CT) from November 2016 to January 2017. The study was approved by the Institutional Review Board at Waterbury Hospital. The objectives of this study were to (1) assess the acceptance of a novel patient garment with increased bodily coverage during colonoscopy and (2) determine patients' perception of embarrassment and physical privacy during colonoscopy.

**Figure 2:**
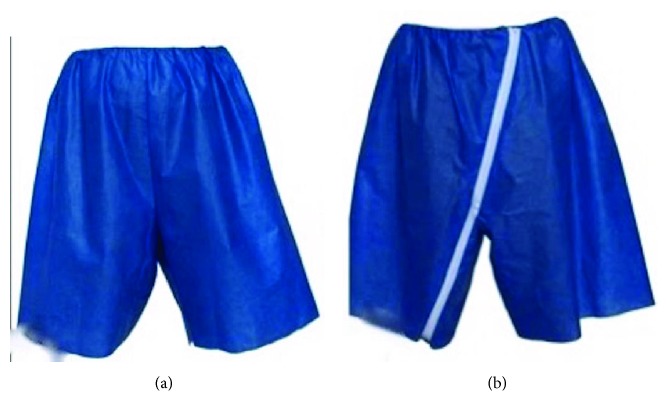
(a) Front view of the Privacy Pants used in this study (reprinted with permission from Dignity Garment LLC). (b) Back view of the Privacy Pants used in this study (reprinted with permission from Dignity Garment LLC).

**Figure 3:**
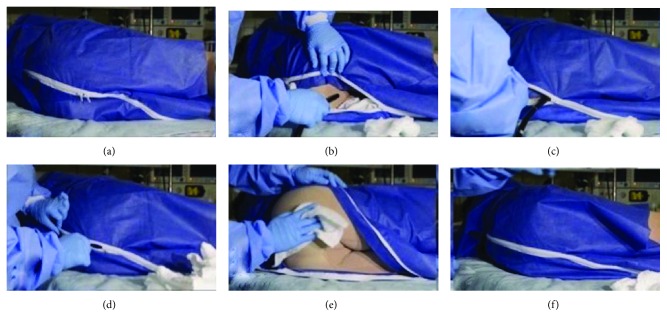
(a) Illustrative image showing the utilization of the novel gown (Privacy Pants): the patient wearing the gown is in left lateral position before the procedure. The double zippers are above the perianal area (reprinted with permission from Dignity Garment LLC). (b) Illustrative image showing the utilization of the novel gown (Privacy Pants): the endoscopist opens the double zipper, performs a perianal examination, applies lubricant, and inserts the endoscope (reprinted with permission from Dignity Garment LLC). (c) Illustrative image showing the utilization of the novel gown (Privacy Pants): during colonoscopy, the endoscopist can close the zippers around the shaft of the endoscope or leave them open (reprinted with permission from Dignity Garment LLC.) (d) Illustrative image showing the utilization of the novel gown (Privacy Pants): the endoscopist or staff can close the double zippers after the procedure is finished (reprinted with permission from Dignity Garment LLC). (e) Illustrative image showing the utilization of the novel gown (Privacy Pants): the double zippers can be completely opened for patient cleaning if needed (reprinted with permission from Dignity Garment LLC). (f) Illustrative image showing the utilization of the novel gown (Privacy Pants): completely zipped garment at procedure conclusion (reprinted with permission from Dignity Garment LLC).

All patients presenting for an outpatient colonoscopy during the study period were offered to utilize the novel garment during colonoscopy (Figures 2 and 3) (Privacy Pants®, Dignity Garment, Madison, MS, USA). All patients received anesthesia-administered sedation, typically with IV Propofol. A structured questionnaire was prepared to address previously reported factors that impact privacy concerns and anxiety before and during colonoscopy. Before colonoscopy, patients were asked to rate their concerns about physical privacy and embarrassment due to bodily exposure on a numerical scale of 0 to 10, with 0 being the lowest and 10 being the highest. After the colonoscopy, patients were asked to rate their satisfaction and experience with the novel garment and how likely they would choose it for future colonoscopy procedures, with all responses utilizing the same numerical scale. They also rated how respected they felt during the procedure and their sense of physical privacy and embarrassment (Table 1).

### 2.1. Statistical Analysis

SPSS version 20 was used for data analysis. Frequencies and percentages were calculated for categorical variables, including age and gender. Means were calculated for the variables scored on a scale of 0 to 10. Age was categorized as less than or equal to 50 and more than 50 years. Continuous variables were compared across age and gender groups by using a *t*-test and Mann-Whitney test where appropriate. *P* value <0.05 was considered significant for all comparisons.

## 3. Results

A total of 120 patients participated in the study, and 54% were female (Table 2). One-fifth of the study population (19.2%) was less than 50 years of age, and 83% of patients were white. 51 respondents (42.5%) had a high school education and 49 (40.8%) had college education. 67% (80) of the subjects had a prior colonoscopy. The indication for colonoscopy was colon cancer screening or surveillance in 68.8% of patients.

The average score was 6.8 when patients were asked whether they felt that the novel garment provided physical privacy during colonoscopy. The average score for satisfaction with the novel gown was 8.3. “Feeling respected during the procedure,” was associated with a mean score of 9.4. 71% of subjects did not feel embarrassed at all during the procedure (score of zero), with an average score of 2.0. 59% (70) of patients said that they will “definitely” (score of 10/10) choose a novel gown for their next colonoscopy, with a mean of 7.8 for all patients. Participants were very agreeable to have a repeat colonoscopy if recommended by their doctors, with a mean score of 9.4. Among the 80 patients who had a prior colonoscopy, when asked to compare their past experience with the traditional gown to the novel gown, the majority (76%) felt that the novel gown increased privacy (mean score of 7.6) and was associated with increased physical privacy (average score 6.8).

### 3.1. Impact Based on Gender

Women were more likely to be concerned about physical privacy during colonoscopy than men (mean score 5.1 vs. 2.6, *P* value <0.05) (Table 1), and women were more concerned regarding embarrassment due to bodily exposure (mean score 5.4 vs. 1.9, *P* value <0.05). Although the average score for embarrassment during colonoscopy was only 2, women had a significantly higher average embarrassment score than men (mean score 3.4 vs. 0.5, *P* value <0.05). Men and women equally felt respected during the procedure, and both felt that the novel gown increased their physical privacy during the procedure (6.8).

### 3.2. Impact Based on Race

83% of participants were Caucasian. Whites were compared with other ethnicities (Table 3). Nonwhites were more likely to be concerned about physical privacy during colonoscopy (mean score 5.4 vs. 3.7, *P* value 0.04). Nonwhites were more likely to have their concern for embarrassment addressed by using a novel gown (mean score 6.7 vs. 4.5, *P* value 0.02). Nonwhites were more likely to report that the novel garment increased physical privacy during colonoscopy (mean score 8.4 vs. 6.1, *P* value <0.05). Nonwhites felt more respected during colonoscopy compared to whites (mean score 9.9 vs. 9.3, *P* value <0.05); however, both groups had high average scores. Nonwhites had higher satisfaction scores for the novel gown (mean score 9.7 vs. 7.8, *P* value <0.05). Nonwhites were more likely to say that they will request the same type of garment for their next colonoscopy (mean score 9.3 vs. 7.5, *P* value <0.05). There was no difference in patients' experiences regarding the novel gown with respect to age or having their first colonoscopy.

## 4. Discussion

Colonoscopy is proven to reduce colorectal cancer incidence and mortality. Due to the limitations of existing hospital gowns, the buttocks and genitals are often exposed during the procedure. Endoscopy teams must take great care to maintain patients' privacy and dignity and reassure patients that privacy will be maintained [1, 13, 14]. Currently, the colon cancer screening rate in the United States is approximately 65% [6]. Socioeconomic status, access to health care, cultural attitudes, religious beliefs, and communication barriers have been shown to influence screening rates [6]. Embarrassment, cited by 35% of people aged 50 to 79 years, was the second most common reported reason for not having a colonoscopy [7]. Utilization of a procedure garment that increases patients' sense of privacy and willingness to have repeat procedures has a potential to increase colonoscopy acceptance and improve colon cancer screening rates.

We sought to evaluate patient perceptions of colonoscopy while utilizing a novel gown designed to increase bodily coverage (Privacy Pants, Jackson, MS, USA). (Figures 2 and 3). We estimate the incremental cost of the novel gown relative to a traditional gown to be less than five dollars. Two-thirds of our study subjects (80) had a prior colonoscopy, and of these, 76% would request the novel gown over a traditional gown for future procedures. The novel gown was associated with physical privacy (average score 6.8), a sense of respect during the procedure (9.4), and high overall satisfaction (8.3).

Seeff et al. reported that women are less likely to have colonoscopy for colorectal screening compared to men and embarrassment is a contributing factor [15, 16]. Of 202 women undergoing colonoscopy, 43% preferred a female endoscopist, and of these, 87% would be willing to wait 30 days for a female endoscopist and 14% would be willing to pay more [8]. Embarrassment was the most common reason for this gender preference. In the United States, 87% of the practicing gastroenterologists are male and only 13% are female [9, 10]. We found that women were more concerned about embarrassment due to bodily exposure during their procedure than men (mean score 5.42 vs. 1.91, *P* value <0.01). Women reported a slightly greater sense of privacy protection during colonoscopy than men with the novel procedure garment (mean score 8.07 vs. 7.15, *P* value 0.27). Below-average colon cancer screening rates have been recognized in African Americans and Asian Americans/Pacific Islanders with embarrassment as a significant barrier to screening [17–19]. Nonwhite participants felt the novel gown minimized their embarrassment during colonoscopy to a greater degree than whites (mean score 6.67 vs. 4.47, *P* value <0.05) and reported an increase in physical privacy (mean score 8.45 vs. 6.12, *P* value <0.05). While the novel garment was favored for use for future colonoscopies by all participants (mean score 7.79), nonwhite participants were more likely to request the novel garment (mean score 9.33 vs. 7.45, *P* value <0.05).

### 4.1. Limitations

The survey questionnaire responses were anonymous; however, responses were self-reported and we recognize the possibility of social desirability bias. Participation in the study was voluntary, thus creating potential selection bias. We estimate that 10% of eligible participants declined participation, although data regarding this group was not recorded. Questions were designed to determine the impact of the novel gown on privacy concerns before and during colonoscopy; however, we recognize that additional factors may potentially influence patient responses. A direct comparison between the novel gown and the traditional gown was not performed, and a comparative study in a larger population with inclusion of groups with low rates of colon cancer screening is warranted.

## 5. Conclusion

Embarrassment is a significant colonoscopy screening barrier. The traditional gown offers limited bodily coverage. We found that all participants, and particularly women and nonwhite participants, reported high rates of respect and satisfaction and decrease in embarrassment utilizing the novel gown during colonoscopy. Patients who previously had colonoscopy with a traditional gown preferred the novel procedure garment with increased bodily coverage. A novel procedure gown may enhance colonoscopy acceptance by minimizing embarrassment, and additional comparative studies are warranted.

## Figures and Tables

**Table 1:** Comparison of males and females.

Question	Mean			*P* value
	All patients	Male	Females	
How concerned are you about your physical privacy during colonoscopy?	3.99	2.58	5.11	<0.01
How concerned are you about embarrassment due to bodily exposure during the procedure?	3.84	1.91	5.42	<0.01
How will the presence of undergarment pants change embarrassment concerns?	4.86	3.55	5.88	<0.01
Do you feel these pants increase physical privacy in colonoscopy?	6.52	6.06	6.87	0.21
How satisfied are you with the pants you had during the procedure?	8.26	8.37	8.17	0.60
How respected did you feel during the procedure?	9.43	9.53	9.34	0.24
How would you rate any feeling of embarrassment during the procedure?	2.03	0.47	3.36	<0.01
Will you request the same type of pants for your next colonoscopy?	7.79	7.50	8.00	0.40
What is the likelihood that you will have this procedure again if your doctor recommended it?	9.47	9.39	9.52	0.94
If you had colonoscopy before, do you feel this type of pants increases physical privacy compared to a traditional gown?	7.66	7.15	8.07	0.27

**Table 2:** Basic demographics.

Variable	Variable category	*N* (%)
Gender (*n* = 120)	Male	55 (46)
	Female	65 (64)
Age (years)	<50	23 (19.2)
	≥50	97 (80.8)
Ethnicity	White	99 (82.5)
	Nonwhite	21 (17.5)
Education	High school	51 (42.5)
	College	49 (40.8)
Colonoscopy indication	Screening	77 (64.2)
	Other	35 (29.2)
History of colonoscopy	First time	39 (32.5)
	More than 1	80 (66.7)

**Table 3:** Comparison of white and nonwhite patients.

Question	Mean			*P* value
	All patients	Male	Female	
How concerned are you about your physical privacy during colonoscopy?	3.99	5.43	3.67	0.04
How concerned are you about embarrassment due to bodily exposure during the procedure?	3.84	5.05	3.57	0.90
How will the presence of undergarment pants change embarrassment concerns?	4.86	6.67	4.47	0.02
Do you feel these pants increase physical privacy in colonoscopy?	6.52	8.45	6.12	<0.01
How satisfied are you with the pants you had during the procedure?	8.26	9.71	7.95	<0.01
How respected did you feel during the procedure?	9.43	9.9	9.33	0.04
How would you rate any feeling of embarrassment during the procedure?	2.03	2.10	2.02	0.96
Will you request the same type of pants for your next colonoscopy?	7.79	9.33	7.45	<0.01
What is the likelihood that you will have this procedure again if your doctor recommended it?	9.47	9.67	9.42	0.69
If you had colonoscopy before, do you feel this type of pants increases physical privacy compared to a traditional gown?	7.66	9.54	7.33	0.02

## Data Availability

The data used to support the findings of this study are available from the corresponding author upon request.
